# Sensorineural hearing dysfunction after discharge from critical care in adults: A retrospective observational study

**DOI:** 10.1016/j.joto.2021.01.001

**Published:** 2021-01-09

**Authors:** Takashi Fujiwara, Mizuki Sato, Shin-ichi Sato, Toshio Fukuoka

**Affiliations:** a Department of Public Health Research, Kurashiki Clinical Research Institute, Miwa 1-1-1, Kurashiki City, Okayama Prefecture, 710-8602, Japan; b Department of Otolaryngology Head and Neck Surgery, Kurashiki Central Hospital, Miwa 1-1-1, Kurashiki City, Okayama Prefecture, 710-8602, Japan; c Department of Critical Care and Emergency Medicine, Kurashiki Central Hospital, Miwa 1-1-1, Kurashiki City, Okayama Prefecture, 710-8602, Japan; d Emergency and Critical Care Center, Kurashiki Central Hospital, Miwa 1-1-1, Kurashiki City, Okayama Prefecture, 710-8602, Japan

**Keywords:** Hearing loss, Critical care, Intensive care, Adverse effects, Observational study

## Abstract

**Background:**

Patients undergoing intensive care are exposed to risk factors for hearing impairment. This study assessed the worse changes in pure tone average (PTA) thresholds after intensive care and identified the factors associated with worse hearing function.

**Methods:**

We conducted a single-centre retrospective study, and included adult patients admitted to the intensive care unit (ICU) of Kurashiki Central Hospital between January 2014 and September 2019, who had regular pure tone audiometry performed before and after ICU admission. Correlations between changes in PTA threshold and patient characteristics, were evaluated. The included ears were classified as those with worse hearing (>10 dB increase in the PTA threshold) and those without worse hearing, and the baseline characteristics were compared.

**Results:**

During the study period, 125 ears of 71 patients (male:female ratio, 35:36; mean age, 72.5 ± 12.3 years) met the eligibility criteria. Age, sex, and the use of furosemide were not correlated with changes in PTA threshold. Univariate analysis showed that ears with worse hearing were associated with a lower serum platelet count than ears without worse hearing (153 ± 85 × 10^9^/L vs. 206 ± 85 × 10^9^/L, respectively; P = 0.010), and the rate of planned ICU admission (elective surgery) was higher in the worse hearing group (57.1% vs. 28.8%, respectively; p = 0.011).

**Conclusions:**

Age, sex, and the use of furosemide did not have adversely affect hearing function. Low serum platelet count and planned admission appear to be risk factors for worse hearing.

## 1 Background

Patients in an intensive care unit (ICU) setting are exposed to some of the risk factors for sensorineural hearing dysfunction (Hamill-Ruth et al., 2003). Loop diuretics, which are well-known ototoxic medications (Ho et al., 2006; Ding et al., 2016), are frequently prescribed to patients in the ICU (de Louw et al., 2015). Unstable conditions, including dehydration and dysrhythmia, also cause sensorineural hearing dysfunction (Luan et al., 2019; Schmutzhard et al., 2013). Previous studies have evaluated hearing dysfunction in neonatal intensive care units, and audiological screening is performed routinely in these patients (Wang et al., 2008; Bonfils et al., 1992). However, few studies have assessed the adverse effects on hearing function in adult ICU patients.

Admission to the ICU is generally unexpected, and it is difficult to obtain a pre-ICU admission hearing function test; in turn, this makes it difficult to assess whether hearing dysfunction after discharge from the ICU is new. Hamill-Ruth et al. conducted a screening protocol in patients admitted to the surgical ICU, and reported that 58.4% of the patients had auditory impairment based on distortion product otoacoustic emission (DPOAE) (Hamill-Ruth et al., 2003). However, their study did not include pre-ICU admission hearing function tests, and it was unclear whether the patients had pre-existing or new-onset hearing dysfunction that occurred in the ICU.

Patients in the ENT department regularly undergo audiometry for disease monitoring. Some of our patients were admitted to the ICU, and the adverse effects of intensive care on hearing function could be evaluated based on changes in audiometry between pre- and post-ICU admission. Therefore, we conducted a retrospective pilot study to assess the adverse effects of intensive care on hearing function using the data from these patients.

## 2 Methods

This single-centre retrospective observational study assessed changes in hearing function in patients undergoing intensive care. We hypothesized that intensive care would have negative effects on hearing function. We also evaluated the patient characteristics associated with worse hearing.

### 2.1 Study population

This study was conducted at Kurashiki Central Hospital, an urban tertiary hospital that serves 800,000 people in the western area of Okayama Prefecture, Japan. There are approximately 70,000 visits to the hospital emergency department annually. Kurashiki Central Hospital has five ICU units (ICU, emergency ICU, coronary care unit-cardiology, coronary care unit-surgery, and the stroke care unit [SCU]). To avoid effects of intracranial disease, we included all ICUs except the SCU. Patients were included if they were ≥20 years old, were admitted to the ICU between January 2014 and September 2019, and had audiometry within 5 years before and after ICU admission. Hearing impairment is usually classified as conductive or sensorineural. Conductive hearing loss is treated medically or surgically (e.g., eardrum ventilation for effusion, ossicle chain reconstruction), and about 40% of patients in the ICU setting have transient middle ear effusion (Hamill-Ruth et al., 1998, 2003). We assessed the adverse effects of intensive care on the inner ear, and therefore excluded ears with conductive hearing loss (e.g., auditory canal edema and otitis media effusion). We also excluded ears with active otological disease that would change the pure tone audiometry results, as well as ears that were deaf before ICU admission.

### 2.2 Measurement of hearing function and data collection

Pure tone averages (PTAs) were obtained via pure tone audiometry before and after ICU admission. Audiometry was performed in a soundproof room according to the standard protocol of the Japan Audiological Society and related international standards (Matsuhira 2009). According to published guidelines (Gurgel et al., 2012; Committee on Hearing and Equilibrium 1995), PTAs were calculated using 0.5-, 1-, 2-, and 4-kHz air conduction thresholds. Data on patient characteristics at ICU admission and the otological findings (e.g., otoscopy and audiometry) were also collected retrospectively by chart review.

We collected data on patient age at ICU admission, as well as ex, body mass index, comorbidities (hypertension and diabetes), length of ICU stay, Sequential Organ Failure Assessment (SOFA) score-related variables (use of vasopressor drugs, PaO2/FiO2 ratio, serum platelets, serum bilirubin, Glasgow Coma Scale), and type of admission (unplanned [emergency] or planned [elective surgery]). The SOFA score assesses the function six organ systems, i.e. the respiratory, cardiovascular, hepatic, coagulation, renal and neurological systems, and the worst value in a 24-h period were collected.

### 2.3 Statistical analysis

Baseline variables with normal distributions are reported as the mean and standard deviation (SD), and those with a skewed distribution are reported as the median and interquartile range (IQR). To assess the correlations between the changes in PTA thresholds and patient characteristics, we used Pearson’s correlation test for age and the dose of furosemide, and Student’s t-test for sex. To identify factors with adverse effects on hearing function, we classified ears as ears with worse hearing (>10 dB increase in the PTA threshold) or ears without worse hearing. The two groups were compared using Student’s t-test and the chi-square test. The institutional review board of Kurashiki Central Hospital approved this study (No 3433).

## 3 Results

### 3.1 Patient characteristics and changes in PTA thresholds

**Fig. 1:**
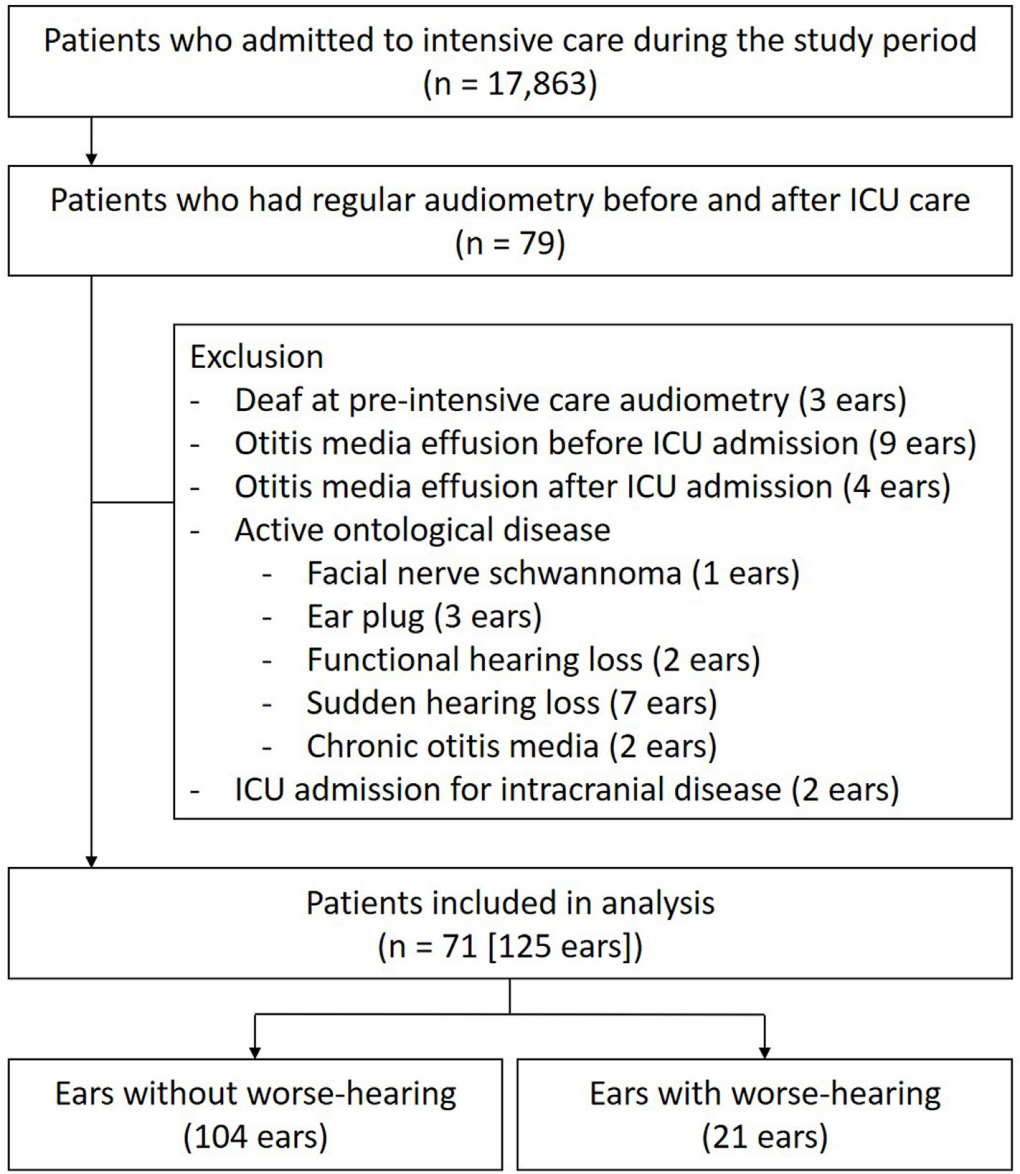
Flow diagram of patients’ selection. During the study period, 17,863 patients had intensive care, and the chart review identified 79 patients who had pure tone audiometry before and after ICU admission. Of the 79 patients, 125 ears from 71 patients were included in analysis, and the reasons for exclusion were shown.

During the study period, 17,863 adult patients were admitted to the included ICUs, and 79 patients met the eligibility criteria. Of these 79 patients, 125 ears of 71 patients (male:female ratio, 35:36; mean age, 72.5 ± 12.3 years) met the inclusion criteria; Fig. 1 provides a flow diagram. The baseline characteristics of the included ears are shown in Table 1. The reasons for ICU admission were infectious disease (n = 7), bleeding (n = 9), metabolic disease (n = 7), cardiovascular disease (n = 14), postoperative management (n = 26), pulmonary disease (n = 2), trauma (n = 1), and others (n = 5). Audiometry was performed a median of 298 days (IQR 54–733 days) before ICU admission and a median of 67 days (IQR: 14–237 days) after ICU discharge. The reasons for audiometry were routine testing (e.g. routine health check-up) (n = 70), audiological complaints (e.g. tinnitus), and enrolment in another clinical trial (n = 45).

**Table 1:** Baseline characteristics of the patients included in the study.

Variables	
Number	71
Age at ICU admission (mean, SD)	72.5 ± 12.3
Sex (male/female)	58/67
Body mass index (mean, SD)	23.6 ± 5.07
Comorbidities	
Hypertension (number, %)	45 (63.4%)
Diabetes (number, %)	18 (25.4%)
SOFA score (mean, SD)	2.70 ± 2.42
Length of stay in ICU (median, range)	2 (1–16)
Length of stay in hospital (median, range)	17 (4–71)
Types of ICU admission	
Planned admission (elective surgery) (number, %)	25 (35.2%)
Emergency (number, %)	46 (64.8%)
Number of ears	125
PTA before ICU admission (dB)	
≤30 (number, %)	41 (32.8%)
>30 (number, %)	84 (67.2%)

ICU, intensive care unit; PTA, pure tone average; SD, standard deviation; SOFA, Sequential Organ Failure Assessment.

**Fig. 2:**
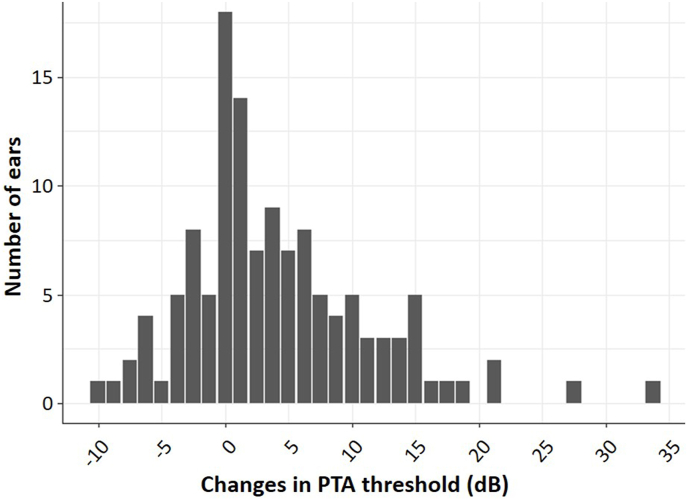
Number of ears by the change in PTA thresholds.

The details of the changes in PTA threshold were as follows: 45 ears had a ≥ −10 dB and ≤0 dB change in the PTA, 59 ears had a >0 dB and <10 dB change in the PTA, 17 ears had a >10 dB and ≤20 dB change in the PTA, and four ears had a >20 dB change in the PTA (Fig. 2).

### 3.2 Sex, age, and, changes in PTAs

**Fig. 3:**
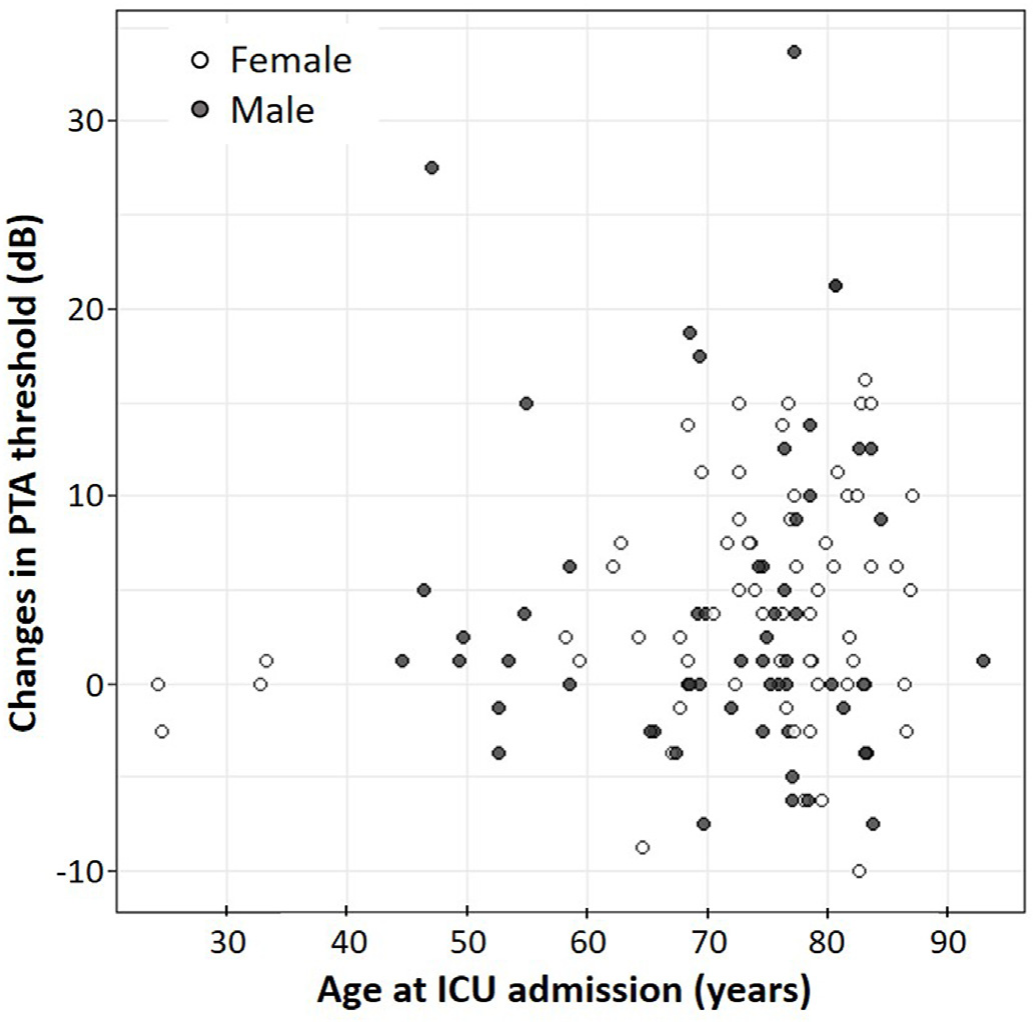
The change in PTA thresholds and age at ICU admission.

The mean change in PTA threshold was 4.14 ± 5.93 in women and 4.03 ± 8.60 in men. The mean difference was 0.11 (95% CI −2.55 to 2.77), which was not statistically significant (p = 0.934). Changes in PTA threshold tended to be larger in older patients, but there was no significant correlation with age (r = 0.091, p = 0.311) (Fig. 3).

### 3.3 Ototoxic medications and changes in PTAs

**Fig. 4:**
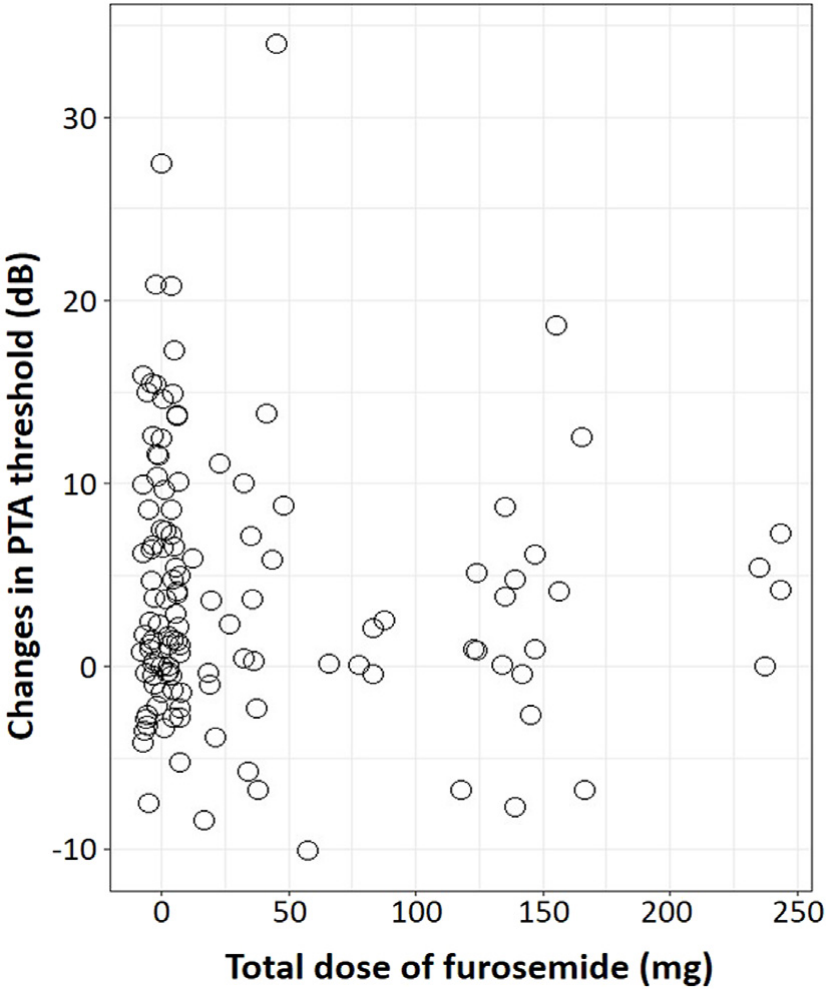
The change in PTA thresholds and dose of furosemide.

The total dose of furosemide during the ICU stay and changes in PTA threshold are shown in Fig. 4. The dose of furosemide was not correlated with changes in PTA threshold. Aminoglycosides are also ototoxic medications (Hong et al., 2020), but no patients included in this study were treated with these agents.

### 3.4 Patient characteristics associated with worse hearing

The modal value of the change in PTA threshold was 0 dB, and changes were mostly in the range of −10 to 10 dB. Therefore, we defined worse hearing as a >10 dB increase in the PTA threshold after ICU admission. We compared the ears with worse hearing and those without worse hearing (Table 2). Of the 125 included ears, 21 (16.8%) had worse hearing. Age at ICU admission and sex were similar between the two groups. The serum platelet count was lower in the ears with worse hearing than ears without worse hearing (153 ± 85 × 109/L vs. 206 ± 85 × 109/L, respectively; p = 0.010), but the SOFA score was similar between the two groups (2.76 ± 2.07 vs. 2.77 ± 2.54, respectively; p = 0.990).

**Table 2:** Comparison between ears with and without worse hearing.

	Ears without worse hearing	Ears with worse hearing	*P*-value
Age at ICU admission (years) (mean, SD)	71.9 ± 12.9	74.7 ± 9.4	0.350
Male (number, %)	47 (45.2%)	11 (52.4%)	0.717
Body mass index	23.3 ± 4.6	24.9 ± 7.8	0.207
PTA before ICU admission	41.9 ± 20.9	42.2 ± 16.2	0.944
Comorbidity			
Hypertension (number, %)	63 (60.6%)	17 (81.0%)	0.086
Diabetes (number, %)	27 (26.0%)	8 (38.1%)	0.291
ICU stay (days) (mean, SD)	3.36 ± 3.62	2.67 ± 3.24	0.212
SOFA score	2.77 ± 2.54	2.76 ± 2.07	0.990
Use of vasopressor drugs (number, %)	16 (15.4%)	3 (14.3%)	0.898
PaO_2_/FiO_2_ ratio	324.0 ± 134.0	377.0 ± 134.5	0.105
Serum platelets ( × 10^9^/L)	206 ± 85	153 ± 85	0.010
Serum bilirubin (mg/dL)	0.90 ± 1.73	0.59 ± 0.42	0.415
Glasgow Coma Scale score ≤ 14 (number, %)	13 (13.4%)	8 (28.6%)	0.083
Serum creatinine (mg/dL)	1.74 ± 2.30	1.30 ± 1.01	0.393
Type of admission			0.011
Unplanned (emergency) (number, %)	74 (71.2%)	9 (42.9%)	
Planned (elective surgery) (number, %)	30 (28.8%)	12 (57.1%)	

ICU, intensive care unit; PTA, pure tone average; SOFA, Sequential Organ Failure Assessment.

**Fig. 5:**
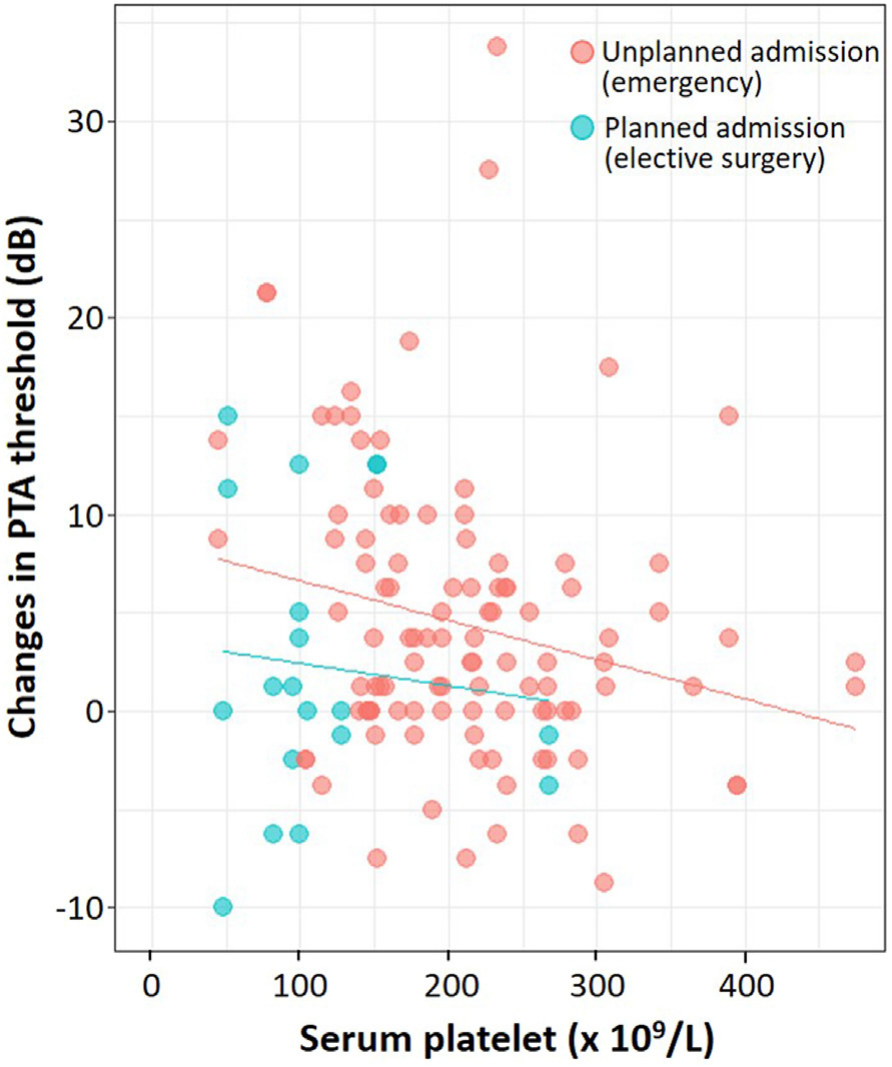
The change in PTA thresholds, serum platelet, and type of admission.

The rate of planned admission was higher in the ears with worse hearing, and the incidence of worse hearing was about threefold higher in ears with planned admission compared to emergency admission (28.6% [12/42] vs. 10.8% [9/83], respectively). The reasons for planned admission were thoracic surgery (33 ears) and others (9 ears), which had respective changes in PTA threshold of 4.51 ± 8.33 and 3.33 ± 8.17. The association of the change in PTAs, serum platelet, and type of admission are shown in Fig. 5.

## 4 Discussion

The results in this study indicated that hearing function was not damaged by intensive care in most cases, although 16.8% of the ears had worse hearing (>10 dB increase in the PTA threshold after discharge from the ICU). Low platelet count and planned admission (elective surgery) were associated with worse hearing outcomes.

It has long been known that intensive care can have adverse effects on hearing function, but this was not considered important because of the low survival rate of ICU patients. Improvement in the survival rates of ICU patients has shifted the focus of outcomes in critical care medicine from short-term mortality to long-term health status (Needham et al., 2012). Audiological hearing dysfunction is associated with cognitive impairment, depression, and reduced well-being (Gao et al., 2020; Lin et al., 2013), and so would affect the long-term health status of ICU survivors. In addition, a previous study suggested that hearing impairment is a risk factor for post-intensive care syndrome (PICS). PICS affects at least half of all survivors after intensive care (Pandharipande et al., 2013), and only 38% of patients with hearing impairment show functional recovery within 6 months after ICU admission (Ferrante et al., 2016).

Hamill-Ruth et al. conducted an audiological screening program in a surgical ICU, and found that about 58.4% of ears of ICU patients had hearing loss, defined based on DPOAE (Hamill-Ruth et al., 2003). Although hearing worsened in 16.8% of ears in ICU patients, the difference could be explained by differences in measurement timings and hearing loss definitions between the two studies. Hamill-Ruth et al. measured hearing loss on ICU admission, while we performed measurements after discharge from the ICU. ICU patients had transient middle ear effusion (Hamill-Ruth et al., 1998), which would have resulted in a higher rate of hearing loss in the study by Hamill-Ruth et al. compared to the present study. This study defined worse hearing as a >10 dB increase in the PTA threshold, while Hamill-Ruth et al. defined hearing loss as a >30 dB increase in the PTA threshold. The prevalence of hearing impairment increases with age (Goman et al., 2020), and the high rate of hearing loss in the study of Hamill-Ruth et al. could be explained by pre-existing hearing loss (i.e., before ICU admission).

There are a number of uncertainties regarding the mechanisms of hearing loss in the ICU, although several mechanisms have been proposed, including microemboli, perfusion abnormalities, hypercoagulability, and ototoxic drugs (Hamill-Ruth et al., 2003; Ho et al., 2006; Ding et al., 2016; Iriz et al., 2008; Young et al., 1987). In this study, there was no correlation between the dose of furosemide and change in PTA threshold, and the rate of use of vasopressor drugs was similar between ears with and without worse hearing. The SOFA score, which assesses dysfunction in six vital organ systems, was similar between ears with and without worse hearing, although the serum platelet count was lower in ears with worse hearing. As part of the SOFA, the serum platelet count is measured for assessing dysfunction of the coagulation system. The incidence of worse hearing was higher in cases of planned admission than emergency admission. Of the ears with planned admission (elective surgery), 78.6% (33/42) underwent thoracic surgery. Patients with thoracic surgery tend to have abnormal coagulation and associated organ dysfunction (Iriz et al., 2008; Kozek-Langenecker et al., 2013). The significant group difference in serum platelets suggests that dysfunction of the coagulation system (e.g. hypercoagulability and microemboli) could explain the results of this study. The duration of ICU stay was shorter in ears with worse hearing; therefore, the worse hearing would be caused by the condition of the patients, not the environments of ICU.

This study had some limitations. Firstly, we only included patients who underwent routine audiometry pre- and post-ICU admission, so there may have been some selection bias. However, it should be noted that no study has reported an association between routine audiometry and poorer hearing function. Nevertheless, it is unclear whether the results can be extrapolated to the general population. Secondly, we did not record the noise levels in the ICUs. A survey in Japan reported that noise levels in an ICU room exceeded 80 dB (Ito et al., 2018), which could damage patient hearing. However, this study was retrospective and the noise levels in our hospital were not recorded; therefore, we could not evaluate them as a potential risk factor for worse hearing. A longer ICU stay would obviously be associated with longer-duration noise exposure in the ICU, but the length of ICU stay tended to be shorter in ears with worse hearing in this study. Therefore, any role of ICU noise in worse hearing would have been small in this study. Thirdly, we excluded ears with active otological disease, but could not exclude all factors that may have influenced hearing function. Patients may be exposed to a number of risk factors, including environmental and dehydration during the ICU stay, but we could not collect data regarding these factors. Finally, we analyzed PTA data, which were obtained for the first time after ICU admission. This was done because PTA threshold levels decrease with age and we wanted to avoid any influence of factors other than ICU stay. It should be noted that elevation of PTA threshold may be reversible depending on the cause (e.g. furosemide). A longitudinal cohort study with longer observation periods is needed to further assess changes of PTA threshold. Similarly, further well-designed prospective observational studies are required to confirm our findings regarding ICU-related sensorineural hearing dysfunction.

## 5 Conclusion

In conclusion, age, sex, and use of furosemide did not have adverse effects on hearing function after discharge from the ICU. Of the 125 included ears, 21 (16.8%) showed adverse changes in the PTA threshold, and low serum platelet count and planned admission were risk factors for worse hearing after discharge from the ICU.

## Conflict of interest & funding

All authors have no potential conflict of interest (e.g. research grants, honoraria for speaking at symposia, financial support, employment/consultation, support from project sponsor, or position on advisory board).

## Funding

This study was funded by Kurashiki Research Institute (Ohara HealthCare Foundation).

## Declaration of competing interest

Fujiwara T is a researcher in Kurashiki Clinical Research Institute, and this study was funded by Kurashiki Clinical Research Institute (Ohara HealthCare Foundation). The funding is used for correction of English writing. This study was not funded by third parties.

## References

[bib1] Bonfils P., Francois M., Avan P. (1992). Spontaneous and evoked otoacoustic emissions in preterm neonates. Laryngoscope.

[bib2] Committee on Hearing and Equilibrium (1995). Committee on Hearing and Equilibrium guidelines for the evaluation of results of treatment of conductive hearing loss. American Academy of Otolaryngology-Head and Neck Surgery Ffoundation, Inc. Otolaryngol Head Neck Surg.

[bib3] de Louw E.J., Sun P.O., Lee J. (2015). Increased incidence of diuretic use in critically ill obese patients. J. Crit. Care.

[bib4] Ding D., Liu H., Qi W. (2016). Ototoxic effects and mechanisms of loop diuretics. J. Otolaryngol..

[bib5] Ferrante L.E., Pisani M.A., Murphy T.E. (2016). Factors associated with functional recovery among older intensive care unit survivors. Am. J. Respir. Crit. Care Med..

[bib6] Gao J., Hu H., Yao L. (2020). The role of social engagement in the association of self-reported hearing loss and health-related quality of life. BMC Geriatr..

[bib7] Goman A.M., Reed N.S., Lin F.R. (2020). Variations in prevalence and number of older adults with self-reported hearing trouble by audiometric hearing loss and sociodemographic characteristics. JAMA Otolaryngol Head Neck Surg.

[bib8] Gurgel R.K., Jackler R.K., Dobie R.A. (2012). A new standardized format for reporting hearing outcome in clinical trials. Otolaryngol. Head Neck Surg..

[bib9] Hamill-Ruth R.J., Ruth R.A. (2003). Evaluation of audiologic impairment in critically ill patients: results of a screening protocol. Crit. Care Med..

[bib10] Hamill-Ruth R.J., Ruth R.A., Googer K. (1998). Hearing impairment in the ICU. Results of a longitudinal pilot study. Crit. Care Med..

[bib11] Ho K.M., Sheridan D.J. (2006). Meta-analysis of frusemide to prevent or treat acute renal failure. BMJ.

[bib12] Hong H., Dowdy D.W., Dooley K.E. (2020). Aminoglycoside-induced hearing loss among patients being treated for drug-resistant tuberculosis in South Africa: a prediction model. Clin. Infect. Dis..

[bib13] Iriz A., Cagli K., Gocer C. (2008). Effects of open heart surgery on hearing thresholds measured by high frequency audiometry. J. Laryngol. Otol..

[bib14] Ito Y., Kawaguchi T., Umemura H., Sato H. (2018). Survey on a-weighted sound pressure levels and their frequency analysis in intensive care unit. Journal of Tokyo University of Information Sciences.

[bib15] Kozek-Langenecker S.A., Afshari A., Albaladejo P. (2013). Management of severe perioperative bleeding: guidelines from the European Society of Anaesthesiology. Eur. J. Anaesthesiol..

[bib16] Lin F.R., Yaffe K., Xia J. (2013). Hearing loss and cognitive decline in older adults. JAMA Intern Med.

[bib17] Luan C.W., Chang J.J., Hsu C.M. (2019). Risk of sudden sensorineural hearing loss in patients with dysrhythmia: a nationwide population-based cohort study. PloS One.

[bib18] Matsuhira T. (2009). Revision of the Japanese industrial standard（JIS）for audiometers. Audiol. Jpn..

[bib20] Pandharipande P.P., Girard T.D., Jackson J.C. (2013). Study Investigators. Long-term cognitive impairment after critical illness. N. Engl. J. Med..

[bib21] Schmutzhard J., Glueckert R., Pritz C. (2013). Sepsis otopathy: experimental sepsis leads to significant hearing impairment due to apoptosis and glutamate excitotoxicity in murine cochlea. Dis Model Mech.

[bib22] Wang C.J., Elliott M.N., McGlynn E.A. (2008). Population-based assessments of ophthalmologic and audiologic follow-up in children with very low birth weight enrolled in Medicaid: a quality-of-care study. Pediatrics.

